# Residue–Residue Interaction Prediction via Stacked Meta-Learning

**DOI:** 10.3390/ijms22126393

**Published:** 2021-06-15

**Authors:** Kuan-Hsi Chen, Yuh-Jyh Hu

**Affiliations:** 1 College of Computer Science, National Yang Ming Chiao Tung University, Hsinchu 300093, Taiwan; cyberetro@gmail.com; 2 Institute of Biomedical Engineering, National Yang Ming Chiao Tung University, Hsinchu 300093, Taiwan

**Keywords:** protein complex, residue–residue interaction, stacked meta-learning

## Abstract

Protein–protein interactions (PPIs) are the basis of most biological functions determined by residue–residue interactions (RRIs). Predicting residue pairs responsible for the interaction is crucial for understanding the cause of a disease and drug design. Computational approaches that considered inexpensive and faster solutions for RRI prediction have been widely used to predict protein interfaces for further analysis. This study presents RRI-Meta, an ensemble meta-learning-based method for RRI prediction. Its hierarchical learning structure comprises four base classifiers and one meta-classifier to integrate predictive strengths from different classifiers. It considers multiple feature types, including sequence-, structure-, and neighbor-based features, for characterizing other properties of a residue interaction environment to better distinguish between noninteracting and interacting residues. We conducted the same experiments using the same data as previously reported in the literature to demonstrate RRI-Meta’s performance. Experimental results show that RRI-Meta is superior to several current prediction tools. Additionally, to analyze the factors that affect the performance of RRI-Meta, we conducted a comparative case study using different protein complexes.

## 1. Introduction

Proteins are the basis of cellular machinery. They are responsible for cellular functions such as cell signaling, molecular motors, and other biological mechanisms. Most protein functions are based on protein–protein interactions (PPIs). Therefore, understanding the binding or interaction mechanisms helps accelerate research on cellular functions, disease causes, immune responses, and drug designs. PPIs have been studied extensively from the cellular to molecular level [1,2,3]. However, understanding protein functions requires more than knowing proteins that can interact; discovering how the proteins interact by identifying binding interfaces between them provides insights into protein functions. Protein–protein binding interfaces can be recognized using X-ray crystallography [4], nuclear magnetic resonance (NMR) [5], and mutagenesis-based approaches [6]; however, these methods are costly and time consuming, which hinders their applicability to all complexes. Therefore, computational methods have been used to predict protein interfaces as preliminary results for further investigation. Protein interactions are driven by hydrophobic effects [7], electrostatic interactions [8], covalent bonds, and physicochemical principles [9]. However, these properties have not been completely understood, which makes the prediction difficult.

Docking-based methods aim to form a protein complex model for its three-dimensional (3D) structure from which protein–protein binding sites can be identified. Docking can be performed using fast Fourier transform (FFT) called FFT docking [10,11]. Other approaches employ geometric hashing [12] and Monte Carlo search [13] to infer possible protein structures. However, these methods are limited by structural information availability such as resolution [14,15].

The increasing availability of protein 3D structures enables learning protein patterns from existing protein structures. A homologous complex is considered a template to infer protein interfaces [16,17]. A homologous complex can be identified based on sequence or structural similarity. Homology-based methods are comparable to the docking methods, but their performances are limited to the number of template structures known.

Several machine learning approaches have been proposed for interface prediction to overcome the unavailability of high-quality protein structural data [18,19]. They extract features from different sources and learn patterns from these features. They can be categorized into two types based on their purpose. The first is partner-independent binding site prediction [20,21], which determines whether the residue of a given protein interacts with any other protein. The other is partner-specific binding site prediction, which identifies the two amino acids that interact in a specific PPI. It describes how the complex is formed by showing the residues involved in interactions.

Partner-specific methods can be further divided into three classes: sequence-based, structure-based, and mixed methods, according to the features they employ. For example, a sequenced-based method extracts features from sequences and uses an artificial neural network with long short-term memory to predict binding sites [18]. By contrast, structure-based methods only use protein structural information. They use various structural properties of proteins for prediction [22]. Unlike sequence- or structure-based methods, mixed methods use different features to increase the prediction accuracy, such as PAIRpred [19], Graph convolutional neural network (Graph CNN) [23], and convolutional neural network (CNN) [24]. They use both sequence and structure information. Sequence features include residue amino acid type and sequence profiles. Structural features include relatively accessible surface area, secondary structures, half-sphere exposure, protrusion index, and depth index. To predict residue–residue-interactions (RRIs), PAIRpred uses a special-purpose pairwise kernel in a support vector machine; by contrast, Fout et al. [23] and Xie et al. [24] proposed using CNNs.

This paper presents RRI-Meta (RRI-Meta-classifier), which is a tool for RRI prediction based on protein sequences or protein structures. We evaluated RRI-Meta on the complexes listed in docking benchmark 5.0 (DBD 5.0) via leave-one-out cross-validation (LOOCV). Experimental results show that RRI-Meta is superior to several other current RRI predictors for several performance metrics.

## 2. Results

We developed RRI-Meta based on a stacked generalization approach to predict the partner-specific interfaces of PPIs. RRI-Meta is structured as an ensemble of four independent base classifiers and one meta-classifier. We compared RRI-Meta with other RRI prediction methods, following the same methodology in the previous studies [18,19,22,24] to maintain consistency and unbiasedness.

### 2.1. Performance Measure

The performance measure used in the comparison is the area under the receiver operating characteristic curve (AUROC). A receiver operating characteristic (ROC) curve is a technique for visualizing and comparing classifiers based on their performance [25], where a classifier is a mapping from instances to predicted classes. In RRI prediction, an instance is a residue pair, and a predicted class is either interacting (positive) or noninteracting (negative). There are four possible outcomes given a classifier and an instance. If the instance is positive, and the prediction is also positive, it is counted as a true positive (TP); if it is classified as negative, it is counted as a false negative (FN). By contrast, if the instance is negative, and the prediction is also negative, it is counted as a true negative (TN); if it is classified as positive, it is counted as a false positive (FP). Given P positive and N negative instances, we define the true positive rate (TPR) and false positive rate (FPR) as follows.

TPR = TP/P = TP/(TP + FN)(1)

FPR = FP/N = FP/(FP + TN) (2)

An ROC curve is a 2D graph in which TPR is plotted on the *Y*-axis, and FPR is plotted on the *X*-axis. The plot for a classifier that curves more toward the left upper corner suggests a higher prediction performance. To compare the performance between two classifiers, we calculate the area under the ROC curves, and a higher AUROC indicates higher performance. 

### 2.2. Performance Comparison between RRI-Meta and Other RRI Prediction Methods

We compared RRI-Meta with four recent RRI predictors, i.e., PPIPP [18], PAIRpred [19], Graph CNN [23], and CNN [24]. Among these, PAIRpred, Graph CNN, and CNN use sequence and structure information to learn patterns from training data and predict whether there is an interaction between each residue pair in the testing data, whereas PPIPP is a sequence-based method. We followed the standards of previous studies [18,19,23,24] to construct a set of protein interaction interfaces on protein complexes of DBD 5.0. The procedures are specified in the method section. These interfaces are the gold standard in the evaluation process, and they also serve as the positive instances of the machine learning methods in the following experiments.

In the comparisons, all predictors except GCNN were trained and tested on the same dataset, i.e., DBD 5.0, because GCNN was pretrained and pretested on specific datasets, and it cannot be trained or tested on different data in its current settings.

**Figure 1:**
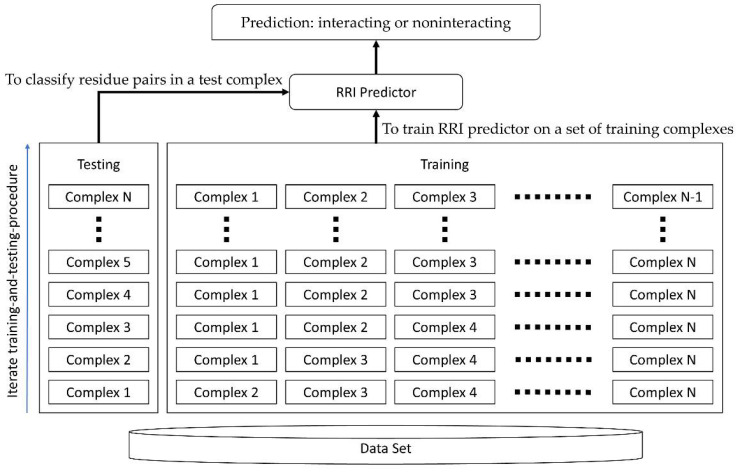
The training–testing process in LOOCV.

**Figure 2:**
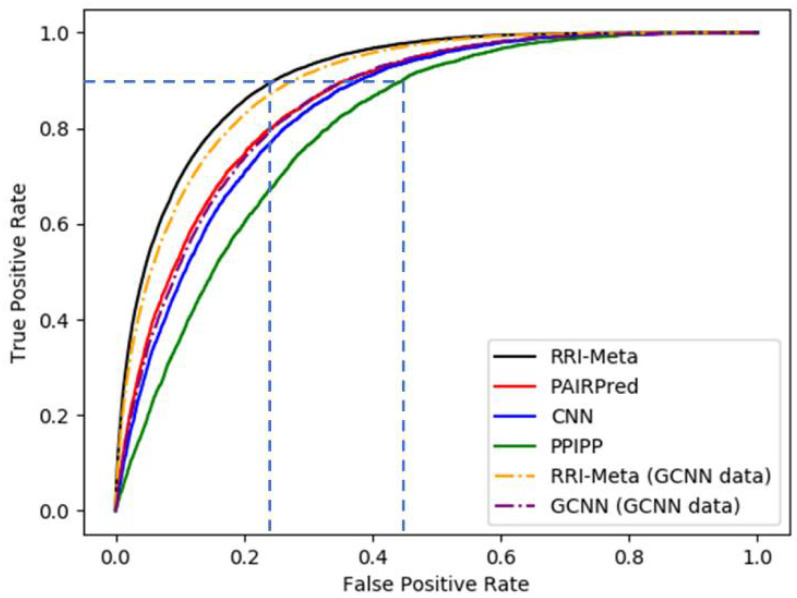
The ROC curves for RRI predictors based on LOOCV.

The protein sequences and protein data bank (PDB) files were downloaded from UniProtKB [26] and RCSB PDB websites [27], respectively to ensure that the test was consistent and unbiased. We conducted LOOCV for all RRI predictors using DBD 5.0 and evaluated their performances based on AUROC. In LOOCV, each complex was reserved for testing the predictive accuracy, and the remaining complexes were used to train the RRI predictors. The same training–testing process was iterated on each protein complex, as illustrated in Figure 1. The final average results are presented in Table 1 and Figure 2.

**Figure 3:**
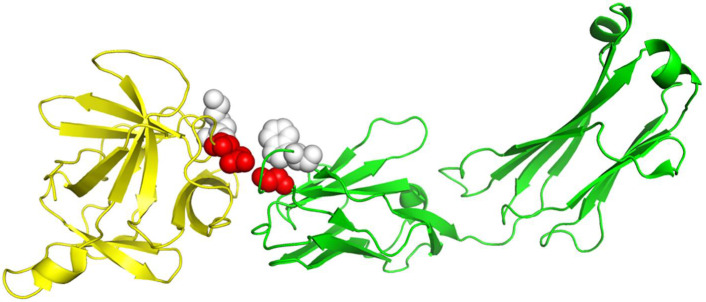
The 3D structure illustration for protein complex 4G6M. Its A chain and L chain are colored in yellow and green, respectively. The red balls indicate the true residue interacting sites, for which RRI-Meta predicted correctly, whereas the predictions of the competing tool, PAIRpred, were incorrect. The gray balls mark the part without residue interactions. RRI-Meta classified it as noninteracting correctly; however, PAIRpred misclassified it to be interacting.

Table 1 shows that RRI-Meta outperformed the other prediction tools in terms of AUROC. In addition, the ROC curve for RRI-Meta shows that it reached 0.9 TPR with a much lower FPR compared with the other predictors (0.25 vs. 0.4), as shown in Figure 2. Due to the special setting of GCNN [23], it was trained on a dataset of 175 complexes and tested on an independent dataset of 55 complexes. To maintain consistency in comparison, we trained and tested RRI-Meta on the same data; the corresponding results are presented in the last two rows of Table 1. With the same but smaller training dataset than DBD 5.0, RRI-Meta also outperformed GCNN on the same test dataset. We use protein complex 4G6M as an example and visualize its 3D structure to demonstrate that RRI-Meta is a more accurate RRI predictor than the other tools. In Figure 3, we show the true interaction interface and the noninteracting part on 4G6M. RRI-Meta was able to classify these sites correctly. By contrast, PAIRpred failed to make the correct predictions. 

### 2.3. Case Study

We conducted a case study to address three factors affecting the performance of RRI predictors, i.e., (a) missing amino acids, (b) feature set, and (c) training complexes, using three protein complexes, 1ML0, 3HMX, and 1RKE, respectively. We show the AUROCs produced by different predictors in the case study in Table 2.

The protein complex 1ML0 comprises of proteins 1DOL (D chain) and 1MKF (A chain). The A chain and D chain of 1ML0 have 382 and 77 residues, respectively, but some are missing. Only 371 and 71 residues can be found in their PDB files, which causes inconsistency between the sequences of PDB and UniProtKB. Current RRI prediction tools retrieve protein sequences from PDB files. By contrast, RRI-Meta obtains sequences from UniProtKB because it is more complete than PDB files.

**Figure 4:**
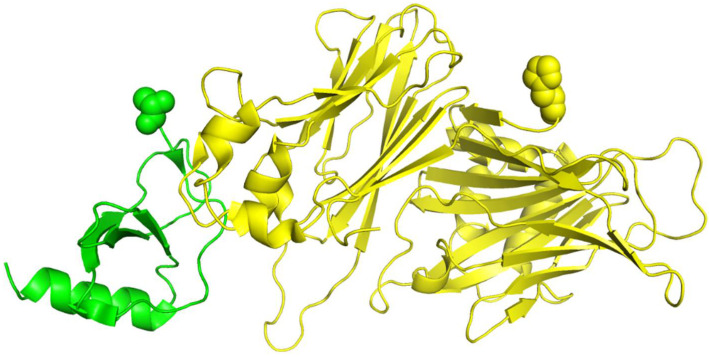
The 3D structure illustration for protein complex 1ML0. Its A and D chains are colored in yellow and green, respectively. A yellow or green ball indicates where some residue(s) should be present but missing.

We show the 3D structures of 1ML0 in Figure 4 based on the sequence stored in PDB. Protein 1DOL is colored in green, and protein 1MKF is colored in yellow. A yellow or green ball indicates where some residue(s) should be present but missing. Consequently, the feature values derived from the sequences stored in PDB for 1ML0 are not the same as those in UniProtKB. To verify the influence of the completeness of protein sequences on RRI prediction, we performed a cross-comparison that tested RRI-Meta using sequences in PDB files and CNN using sequences in UniProtKB. The AUROC of 1ML0 generated by RRI-Meta decreased to 0.71, whereas the AUROC of 1ML0 generated by CNN increased to 0.68. The results suggest that a more complete protein sequence warrants more accurate sequence-based features and consequently improves prediction performance. Further analysis showed that the impact of missing residues is more significant when they are close to interacting residues.

The features used to represent the data strongly affect the machine learning performance. Compared with other RRI predictors in this study, RRI-Meta uses various features to represent protein complexes. In addition to combining sequence-based and structure-based features to utilize the synergy, RRI-Meta also considers neighbor-based features to characterize 1D and 3D proximities. Neighbor-based features describe the surrounding environment of residue pairs providing information that may be the key factors in identifying the interacting residue. To validate the benefit of richer feature representations, we tested RRI-Meta on 3HMX using only the features adopted by PAIRpred and found that the AUROC decreased from 0.97 to 0.94. 

In addition to the features, the protein complexes used to train the predictors are also crucial for prediction performance, especially when the number of noninteracting residues is considerably larger than interacting ones. Protein complexes that are remotely related to the test complex are not appropriate for training predictors. The irrelevancies in these complexes can mislead the learning process of the predictors. By excluding the protein complexes with low similarity to the test example from the training data, we can improve AUROC in prediction. By contrast, we observed a decrease in the AUROC of RRI-Meta from 0.86 to 0.84 for 1RKE without the complex-filtering mechanism. Furthermore, we noted that the complex-filtering mechanism could improve PAIRpred and CNN; their AUROCs increased to 0.83 and 0.85, respectively. These findings indicate the importance of training data selection, and we have embedded a filter in RRI-Meta to address the issue.

### 2.4. Ratio of Positive Data to Negative Data for Training

RRI prediction is a class-imbalanced classification problem in which there are significantly more noninteracting residues than interacting residues in a protein complex. The imbalance between the numbers of noninteractions and interactions in a complex obstructs the predictors from learning the correct concept of RRI even after removing the irrelevant protein complexes from the training dataset. One consequence of using an imbalanced dataset in the training phase is that the classifier would predict most instances as the majority to achieve higher accuracy. For example, if there is one interacting residue pair and 1000 noninteracting ones, the classifier could easily achieve 0.999 accuracy if it classifies all residue pairs as noninteracting. Sampling techniques such as oversampling and undersampling have been commonly used to resolve class imbalance problems. In this work, we used undersampling to mitigate the class imbalance problem.

We conducted the experiments with different ratios between noninteracting and interacting residues because the noninteracting residues can sometimes be 1000 times more than interacting residues in a protein complex, and undersampling the noninteracting residues to the same number of interacting residues may not necessarily reflect the correct class boundary. We analyzed the effects of class ratios on prediction performance. We varied the ratio from 1:1 to 1:6; the corresponding results are given in Table 3.

Table 3 reveals that the AUROC can vary as the ratio changes, but the difference is minor, suggesting that RRI-Meta is relatively stable and insensitive to class ratio settings. Increasing the number of noninteracting residues in training data did not necessarily improve the prediction performance.

### 2.5. Ablation Study of Features

The superiority of RRI-Meta over other predictors in our comparisons is partly attributed to the use of a wider variety of features. Benefiting from the synergy of various features, RRI-Meta can more easily identify the class boundary between noninteracting and interacting residues. To further investigate the importance of different features, we conducted an ablation study to evaluate various feature combinations.

We divided the features into three categories according to their distinct properties: sequence-, structure-, and neighbor-based features. Then, we evaluated the prediction performance of RRI-Meta using six different combinations of feature types; the corresponding results are given in Table 4. Although the differences in prediction performance using different feature combinations were marginal, RRI-Meta achieved the highest AUROC when considering all feature types. In addition, Table 4 reveals that compared with using a single feature type, using a combination of two feature types can improve RRI-Meta’s performance. These findings indicate that different feature types can contribute to RRI prediction differently and complement each other. A good synergetic combination of features can lead to a more accurate RRI prediction.

## 3. Discussion

RRIs play an important role in biological functions. Previous research extracted features from sequence and structure information as inputs for machine learning algorithms and achieved remarkable RRI prediction results. In this research, we developed RRI-Meta, which extends the existing feature set, providing a more detailed residue pair description to the machine learning algorithm, and incorporates a stacked generalization framework with a protein complex filter to predict RRIs. Four weak classifiers, Decision Tree (DT) [28], Naïve Bayesian (NB) [29], Artificial Neural Network (ANN) [30], and Random Forest (RF) [31], were used as base learners at the bottom level of RRI-Meta to generate initial predictive results. LightGBM [32] was selected as the meta-classifier to arbitrate among the base learners for the final prediction. RRI-Meta leverages the advantages of base learners. In this design, RRI-Meta can integrate the predictive strengths of different predictors and find better discriminating patterns from initial results and primitive features. In addition, RRI-Meta uses three feature types to characterize residue interactions better and consequently identify a better boundary between noninteracting and interacting residues in the feature space. Following previous works [18,19,24], we performed LOOCV on RRI-Meta and compared it with other current RRI predictors using the same dataset to maintain consistency and unbiasedness. The results demonstrate that RRI-Meta outperformed the other comparative methods in terms of AUROC.

First, to analyze the factors contributing to the superiority of RRI-Meta, we conducted a case study of three protein complexes. In the case study, the analysis of effects of protein sequence completeness on RRI prediction indicates that a protein sequence with fewer missing amino acids can provide more sequence-based information of the environment in which residue interactions occur. Therefore, prediction tools can benefit from more precise feature descriptions and produce more accurate results. We tested RRI-Meta on PDB sequences and UniProtKB sequences in the same 65 protein complexes with missing residues. The results showed that when extracting features from more complete UniProtKB sequences, RRI-Meta made more accurate RRI predictions than when extracting features from PDB sequences. Second, to verify the contribution of a wider variety of features for prediction performances, we compared the performance of RRI-Meta using the proposed extended features with that using the less versatile features used by other predictors such as PAIRpred. We observed that combinations of multiple feature types enable RRI-Meta to more easily identify the correct boundary between noninteracting and interacting residues, thereby achieving higher prediction performance. Third, to resolve relevant features in training data, we equipped RRI-Meta with a protein complex filter removing residue pairs from complexes that are remotely related to the test complex. We tested the usefulness of the filter by evaluating the prediction performance of several predictors with and without the filter and found that by filtering out irrelevant protein complexes from the training data, the predictors’ performance improved remarkably.

In addition, we conducted an ablation study of feature importance to verify the synergy of different feature types, i.e., sequence-, structure-, sequence neighbor-, and structure-neighbor-based features. We use RRI-Meta to evaluate the effects of all combinations of different features on prediction performance. As expected, different types of features characterized the residue interaction environment differently, and none of the features seemed to dominate the others. Using different combinations of features improves RRI-Meta’s performance compared with using a single feature type, indicating the synergy of these features.

## 4. Materials and Methods

**Figure 5:**
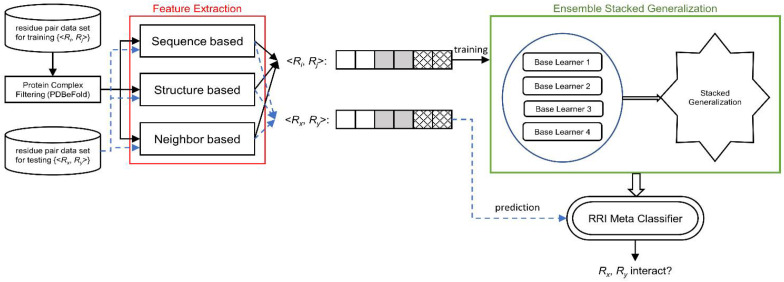
System Diagram of RRI-Meta for predicting residue–residue interactions.

We designed RRI-Meta based on a stacked generalization framework and developed a two-level ensemble meta-learner for RRI prediction. A complete system is implemented, as illustrated in Figure 5. The source code and the data used in the study are available in the Github repository, https://github.com/mlbioinfolab/rrimeta (accessed date: 13 June 2021).

### 4.1. Protein Features

For RRI prediction, features used to describe residue pairs are crucial to the success of machine learning. RRI-Meta uses both sequence- and structure-based protein features. In this study, each residue pair, (*r_i_*, *r_j_*), is represented by a feature vector, <*f_i1_*, *f_i2_*, …, *f_ip_*, *f_j1_*, *f_j2_*, …, *f_jp_*>, where *p* is the total number of features for residues *r_i_* and *r_j_*; *f_in_* and *f_jn_* are the *n-th* features of *r_i_* and *r_j_*, respectively. The feature values can be derived from protein sequences and their PDB files.

#### 4.1.1. Sequence-Based Feature: Physicochemical Property

As the basis for RRI prediction, we characterize each protein based on 12 physicochemical properties of its composite amino acids [33,34,35,36,37,38,39,40]: hydrophilicity, flexibility, accessibility, turns scale, exposed surface, polarity, antigenic propensity, hydrophobicity, net charge index of the side chains, polarizability, solvent-accessible surface area, and side-chain volume. Among these properties, hydrophobicity and polarity are each calculated according to two different scales. The values of 14 physicochemical property scales of 20 essential amino acids are listed in Table 5. We translated each amino acid into a vector of 14 numeric values, corresponding to a physicochemical scale value in Table 5.

#### 4.1.2. Sequence-Based Feature: Sequence Profile

We used PSI-BLAST [41] to extract the position-specific scoring matrix (PSSM) and position-specific frequency matrix (PSFM) of a protein. In different measures, both PSSM and PSFM are good indicators of how better conserved and how more frequent a given amino acid is within the sequence alignment than expected by chance. A high PSSM or PSFM score suggests that an amino acid could be a critical functional residue that is probably an active site for RRI. Additionally, we also considered the conservation index computed by Al2co [42]; it defines the index values from entropy, variance, and alignment matrix score.

#### 4.1.3. Structure-Based Feature: Polypeptide Geometric Property

We used PSAIA to compute solvent-accessible surface score (ASA), relative ASA (RASA), depth index (DPX), and protrusion index (CX) from protein structure files. Both ASA and RASA are influential in protein folding and can affect interacting surfaces. DPX measures the distance between an atom and the closest solvent-accessible atoms in a protein. CX measures the convexity of each nonhydrogen atom in a protein. Similar to ASA and RASA, DPX and CX are potential factors of residue interactions.

#### 4.1.4. Structure-Based Feature: Secondary Structure

Secondary structures are important for residue contact because they demonstrate different contact energies [42,43]. We used the Dictionary of Protein Secondary Structure (DSSP) to determine the secondary structure and presented it using a one-hot encoding scheme for simplicity.

#### 4.1.5. Structure-Based Feature: Half-Sphere Exposure

Half-sphere exposure (HSE) is an alternative measure of protein solvent exposure that shows the convexity of a residue in a protein. We used the BioPython package [44] to calculate the HSE of each residue in PDB files.

#### 4.1.6. Neighbor-Based Feature: Sequence and Structure

**Figure 6:**
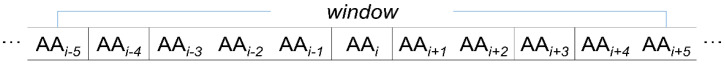
Sequence neighbors of amino acid AA*_i_*.

**Figure 7:**
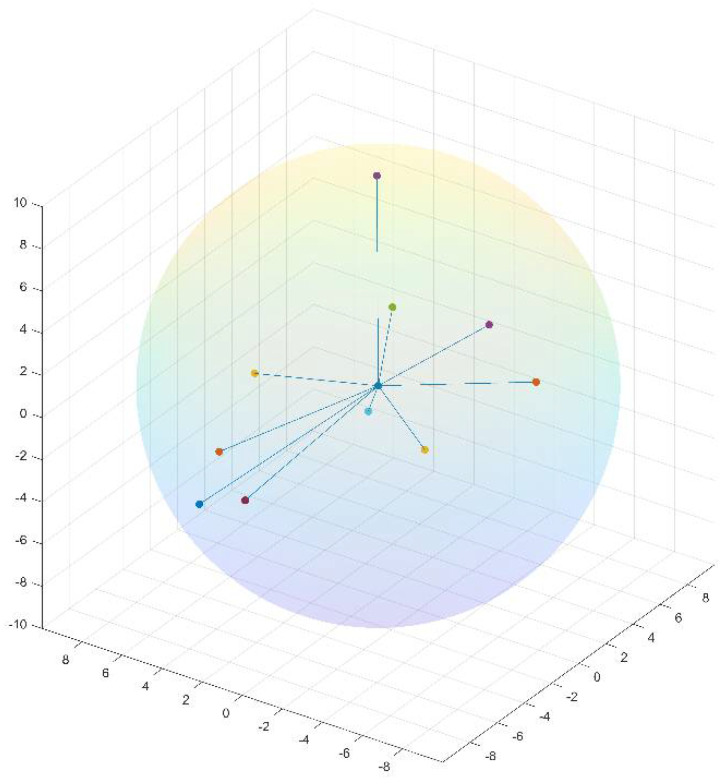
Structure neighbors of a given amino acid AA*_i_* in the 3D space. AA*_i_* is the center of the sphere.

We define the neighbors of a residue based on two aspects: sequence and structure. Sequence neighbors of a residue are the nearest N residues in the sequence. Figure 6 shows an example of the sequence neighbors of amino acid AA*_i_*. A residue and its neighbors can be presented as a window of length N + 1 centered at that residue. Given a window-based neighborhood, for each residue in a protein, we generate its neighbor-based features from its neighbors’ primitive feature values such as physicochemical properties, ASA, and DPX. By contrast, the structure neighbors of a residue are the closest residues in their 3D structures. Figure 7 illustrates the structure neighbors of a given residue AA*_i_* in the 3D space. The distance between two residues is defined as the Euclidean distance between their Cα atoms. To generate structure neighbor-based features for a residue, we only consider its nearest 10 structural neighbors, and their distances from the residue must be less than 10 angstroms (Å). Similar to sequence neighbor-based features, we construct neighbor-based features from these structural neighbors’ feature values. Notably, neighbor-based features can be defined differently depending on neighbors’ feature values. We currently define neighbor-based features as the minimum, maximum, mean, and sum of feature values in a neighborhood.

### 4.2. Complex Filtering

**Figure 8:**
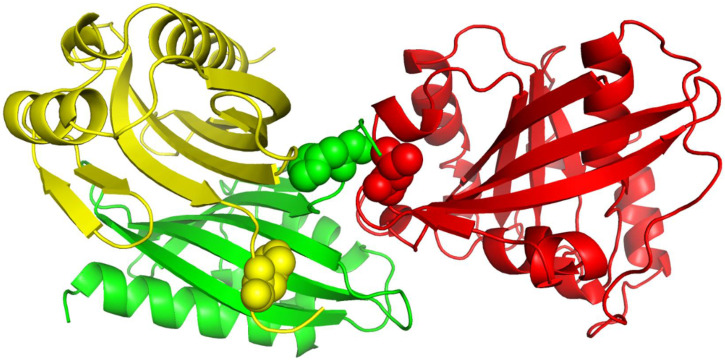
The 3D structure of protein complex 1A2K. The A, B, and C chains are colored in yellow, green, and red, respectively. The green and red balls indicate part of the interaction interface within 1A2K. The yellow balls share the same sequence configuration as the green balls, but they do not interact with the red balls.

The structure of a protein determines its function in biological processes [45]. Two polypeptide chains with the same sequence in the same protein complex can interact with different polypeptide chains on different residue pairs. For example, in Figure 8, the A and B chains of 1A2K have the same sequence configuration, but they interact with the C chain differently because the A chain and the B chain do not fold in exactly the same style in the 3D space. Protein structures shape the residue interaction environment; therefore, a residue interface in a protein complex is expected to be more similar to that in another complex with similar structures rather than random. Based on this conjecture, we prepare the training complexes for RRI-Meta by removing the sufficiently different complexes between the test complex and the training dataset. For each training complex *T_i_* in the training dataset, we calculate its similarity to a test complex *S* by applying PDBeFold [46]. We remove training complexes with similarity scores less than 0.05 and train RRI-Meta on the remaining complexes in the training dataset.

### 4.3. Ensemble Learning by Stacked Generalization

**Figure 9:**
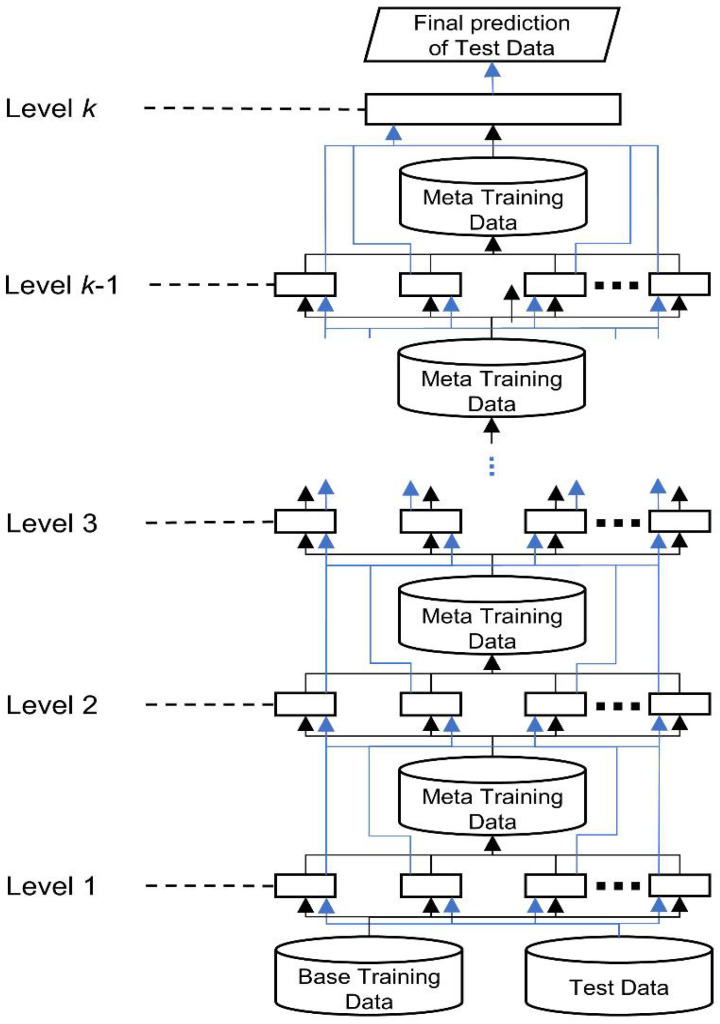
Hierarchical architecture of stacked generalization. The black lines indicate the data flow of training data; the blue lines show the data flow of test data.

Unlike other iterative ensemble learning approaches based on bagging or boosting, stacked generalization [47] works as layered processes to reduce learner bias. In stacked generalization, each of a set of base learners is trained from a dataset, and the predictions of these base learners become the meta features. A successive layer of meta learners receives the meta features as the input to train the meta models in parallel, passing their output to the subsequent layer. A single classifier at the top level makes the final prediction. Stacked generalization is considered a form of meta learning because the transformations of the training data for the successive layers contain the information of the predictions of the preceding learners, which is a form of meta knowledge. Figure 9 shows a generic hierarchical architecture of stacked generalization.

**Figure 10:**
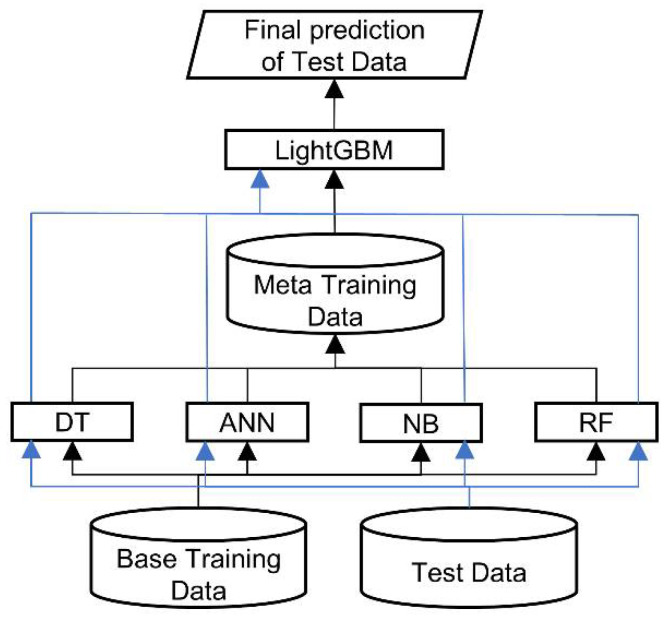
Architecture of RRI-Meta.

This study adopted the stacked generalization strategy to develop a two-level stacking architecture for RRI prediction, as presented in Figure 10. The bottom level comprises four base classifiers: Decision Tree (DT) [28], Naïve Bayesian (NB) [29], Artificial Neural Network (ANN) [30], and Random Forest (RF) [31]. At the top level, we use LightGBM [32] as a meta-classifier that arbitrates among the base classifiers (bottom level), making the final prediction. The predictions of the base classifiers provide the meta-data for training the top level LightGBM. To classify RRI for a new complex, we first feed the feature vector derived from proteins to each trained base classifier, which generates a prediction. Subsequently, the predictions of the four classifiers are input to the trained LightGBM, which makes the final RRI prediction for the new complex.

### 4.4. Dataset

To conduct consistent and unbiased experiments, we followed previous works [19,24] and used the same docking benchmark 5.0 dataset (DBD 5.0) for performance evaluation. DBD 5.0 comprises 224 distinct bound protein complexes and their unbound ligands and receptors, with 3D structures identified via X-ray crystallography.

### 4.5. Definition of Residue Interactions

This study adopted the most commonly used definition of interacting residue pairs [18,19,24]. Two residues are considered to interact if at least one of the distances between their heavy atom pairs is less than or equal to 6.0 Å. Based on this definition, 20,777 interacting and 14,945,106 noninteracting residue pairs are found in DBD 5.0. Each protein complex contains 93 interacting and 67,917 noninteracting residue pairs on average.

## 5. Conclusions

Understanding how the proteins interact by identifying binding interfaces between them provides insights into protein functions. The increasing availability of various protein data has encouraged the development of different computational methods to escalate the study of protein residue interactions. As a result, several machine learning approaches have been proposed for interface prediction, adopting different design philosophies, including learning strategies, predictive models, and data representations.

We introduce RRI-Meta, a new approach for RRI prediction. It employs a hybrid feature representation that combines protein sequence properties, protein structure information, and neighbor-based attributes. In addition to considering a broader variety of protein features to distinguish between noninteracting and interacting residues, unlike current prediction methods, RRI-Meta uses an ensemble meta-learning architecture to benefit from multiple predictive models. The performance of RRI-Meta was extensively compared with those of four state-of-the-art RRI predictors on a benchmark dataset. The experimental results demonstrated a favorable performance over the others.

Additionally, we conducted a case study to analyze three important factors, missing amino acids, feature set, and training complexes. These factors can affect the performance of any RRI predictor. From the case study, we obtained the following findings. First, a more complete protein sequence warrants more accurate sequence-based features and consequently improves prediction performance. Second, richer feature representations characterize residue interaction environments more thoroughly and thus provide a clearer class boundary for predictors to distinguish between interactions and noninteractions. Last, irrelevant or remotely related protein complexes can mislead the learning of RRI predictors. A proper training dataset is crucial to the success of RRI predictors.

Overall, the results of the comparative experiments and the case study verify the feasibility and superiority of the proposed ensemble meta-learning architecture, RRI-Meta, in RRI prediction. As a wider variety of machine learning algorithms becomes available for being base learners or meta learners, the performance of RRI-Meta can be further improved.

## Figures and Tables

**Table 1:** Average results for LOOCV on DBD 5.0.

Method	AUROC
PAIRpred	0.86
PPIPP	0.80
CNN	0.85
RRI-Meta	0.90
GCNN *	*0.86*
RRI-Meta *	*0.89*

* Using a training dataset of 175 complexes and an independent test dataset of 55 complexes for evaluation.

**Table 2:** AUROCs of RRI predictors in case study.

Complex	RRI-Meta	PPIPP	PAIRpred	CNN
1ML0	0.74	0.62	0.72	0.66
3HMX	0.97	0.89	0.93	0.91
1RKE	0.86	0.76	0.81	0.82

**Table 3:** Prediction performances based on different class ratios.

Ratio Interacting/Noninteracting	AUROC
1:1	0.88
1:2	0.89
1:3	0.90
1:4	0.90
1:5	0.89
1:6	0.89

**Table 4:** Results of ablation study on feature importance.

Sequence	Structure	Sequence Neighbor	Structure Neighbor	AUROC
O	X	X	X	0.84
X	O	X	X	0.80
X	X	O	X	0.87
X	X	X	O	0.84
O	O	X	X	0.86
O	X	O	X	0.88
O	X	X	O	0.87
X	O	O	X	0.88
X	O	X	O	0.85
X	X	O	O	0.88
O	O	O	X	0.88
O	O	X	O	0.87
O	X	O	O	0.89
X	O	O	O	0.88
O	O	O	O	0.90

Note. O indicates the features are included; X means the features are excluded.

**Table 5:** Values of the 12 physicochemical property scales of the 20 essential amino acids.

AA	H_11_ *	H_12_ *	H_2_	NCI	P_11_ *	P_12_ *	P_2_	SASA	V	F	A_1_	E	T	A_2_
A	0.62	2.1	−0.5	0.007	8.1	0	0.046	1.181	27.5	−1.27	0.49	15	−0.8	1.064
C	0.29	1.4	−1.0	−0.037	5.5	1.48	0.128	1.461	44.6	−1.09	0.26	5	0.83	1.412
D	−0.9	10.0	3.0	−0.024	13.0	40.7	0.105	1.587	40.0	1.42	0.78	50	1.65	0.866
E	−0.74	7.8	3.0	0.007	12.3	49.91	0.151	1.862	62.0	1.6	0.84	55	−0.92	0.851
F	1.19	−9.2	−2.5	0.038	5.2	0.35	0.29	2.228	115.5	−2.14	0.42	10	0.18	1.091
G	0.48	5.7	0.0	0.179	9.0	0	0	0.881	0	1.86	0.48	10	−0.55	0.874
H	−0.4	2.1	−0.5	−0.011	10.4	3.53	0.23	2.025	79.0	−0.82	0.84	56	0.11	1.105
I	1.38	−8.0	−1.8	0.022	5.2	0.15	0.186	1.81	93.5	−2.89	0.34	13	−1.53	1.152
K	−1.5	5.7	3.0	0.018	11.3	49.5	0.219	2.258	100	2.88	0.97	85	−1.06	0.93
L	1.06	−9.2	−1.8	0.052	4.9	0.45	0.186	1.931	93.5	−2.29	0.4	16	−1.01	1.25
M	0.64	−4.2	−1.3	0.003	5.7	1.43	0.221	2.034	94.1	−1.84	0.48	20	−1.48	0.826
N	−0.78	7.0	2.0	0.005	11.6	3.38	0.134	1.655	58.7	1.77	0.81	49	3.0	0.776
P	0.12	2.1	0.0	0.240	8.0	0	0.131	1.468	41.9	0.52	0.49	15	−0.8	1.064
Q	−0.85	6.0	0.2	0.049	10.5	3.53	0.18	1.932	80.7	1.18	0.84	56	0.11	1.015
R	−2.53	4.2	3.0	0.044	10.5	52.0	0.291	2.56	105	2.79	0.95	67	−1.15	0.873
S	−0.18	6.5	0.3	0.005	9.2	1.67	0.062	1.298	29.3	3.0	0.65	32	1.34	1.012
T	−0.05	5.2	−0.4	0.003	8.6	1.66	0.108	1.525	51.3	1.18	0.7	32	0.27	0.909
V	1.08	−3.7	−1.5	0.057	5.9	0.13	0.14	1.645	71.5	−1.75	0.36	14	−0.83	1.383
W	0.81	−10	−3.4	0.038	5.4	2.1	0.409	2.663	145.5	−3.78	0.51	17	−0.97	0.893
Y	0.26	−1.9	−2.3	117.3	6.2	1.61	0.298	2.368	0.024	−3.3	0.76	41	−0.29	1.161

H_11_ and H_12_: hydrophobicity; H_2_: hydrophilicity; NCI: net charge index of side chains; P_11_ and P_12:_ polarity; P_2_: polarizability; SASA: solvent-accessible surface area; V: volume of side chains; F: flexibility; A_1_: accessibility; E: exposed; T: turns; A_2_: antigenic. * Hydrophobicity (H_11_ and H_12_) and polarity (P_11_ and P_12_) were calculated using two methods.

## Data Availability

The data that support the findings of this study are available from the corresponding author.
